# ROS are required for the germinative cell proliferation and metacestode larval growth of *Echinococcus multilocularis*

**DOI:** 10.3389/fmicb.2024.1410504

**Published:** 2024-06-07

**Authors:** Ye Tian, Zhe Cheng, Defeng Ge, Zhijian Xu, Huijuan Wang, Xiazhen Li, Huimin Tian, Fan Liu, Damin Luo, Yanhai Wang

**Affiliations:** ^1^State Key Laboratory of Cellular Stress Biology, School of Life Sciences, Xiamen University, Xiamen, Fujian, China; ^2^Parasitology Research Laboratory, School of Life Sciences, Xiamen University, Xiamen, Fujian, China; ^3^School of Medicine, Xiamen University, Xiamen, Fujian, China

**Keywords:** *Echinococcus multilocularis*, reactive oxygen species, germinative cells, proliferation, hypoxia-inducible factor 1α

## Abstract

The potentially lethal zoonotic disease alveolar echinococcosis (AE) is caused by the metacestode larval stages of the tapeworm *Echinococcus multilocularis*. Metacestode growth and proliferation occurs within the inner organs of mammalian hosts, which is associated with complex molecular parasite–host interactions. The host has developed various ways to resist a parasitic infection, and the production of reactive oxygen species (ROS) is one of the most important strategies. Here, we found that scavenging of ROS reduced metacestode larval growth and germinative cell proliferation in *in vivo* models. Furthermore, using *in vitro-*cultured metacestode vesicles, we found that increased ROS levels enhanced metacestode growth and germinative cell proliferation, which was achieved by positively activating the ROS-EmERK-EmHIF1α axis. These results indicate that, beside its capacity to damage the parasite, ROS also play critical roles in metacestode growth and germinative cell proliferation. This study suggests that the effects of ROS on parasite may be bidirectional during AE infection, reflecting the parasite’s adaptation to the oxidative stress microenvironment.

## Introduction

*Echinococcus multilocularis* (*E. multilocularis*), one of the platyhelminth parasites, is the causative agent of alveolar echinococcosis (AE) (Wang et al., 2023). Human infection initiates with occasional ingestion of the infective eggs. The eggs hatch in intestine to release oncospheres that subsequently reach liver, where they usually settle down and develop into metacestode vesicles. The metacestode vesicles then grow infiltratively like a tumor in liver and other host organs, eventually leading to organ failure (Wen et al., 2019). When the parasite grows for long time periods in close contact to the inner organs of mammals, the molecular mechanisms of the interaction between parasite and host are highly complex (Brehm and Koziol, 2017).

Parasite–host interaction could be divided into two equal important aspects. Whereas most studies have focused on the effects of parasite on the host (Yasen et al., 2021; Jiang T. et al., 2022; Jiang X. et al., 2022; Wu et al., 2023), relatively few studies have been reported concerning the effects of host on the parasite (Hemer et al., 2014; Cheng et al., 2017). The most important impact of host on the parasite is immune response and the oxidative stress it induces. During the infection, host immune system promptly recruits immune cells and uses macrophages and neutrophils to resist the invading parasites, generating a large amount of toxic reactive oxygen species (ROS), which is called the ‘respiratory burst’ (Tikhomirova et al., 2023). These ROS directly damage the parasites that infect tissues (Wang et al., 2021). Drugs commonly used for AE treatment, albendazole and mebendazole, can stimulate ROS production to enhance oxidative stress (Locatelli et al., 2004; Siles-Lucas et al., 2018). Extensive studies have demonstrated the harms of ROS on parasites. However, emerging evidence show that ROS have been called ‘double-edged swords of life’ in pathogen clearance (Mittler, 2017). Claudia and colleagues found that oxidative stress contributes to *Trypanosoma cruzi* persistence in host tissues (Paiva et al., 2012). Different from intracellular unicellular parasites, *E. multilocularis*, an extracellular multicellular parasite, have more complex interactions with the host. These findings prompt us to reappraise the role of ROS in the growth of metacestode larvae during AE infection.

The larval growth and development of *E. multilocularis* are dominated by the germinative cells, a population of adult stem cells similar to the ‘neoblasts’ of the free-living flatworm planarian (Newmark and Sanchez, 2000). The germinative cells are pluripotent and are the only proliferative cells in the *E. multilocularis* metacestode larvae (Koziol et al., 2014). ROS have been shown to regulate the cellular activities of stem cells (Sies and Jones, 2020). There is a clear correlation of ROS levels in stem cells with their functions (Urao and Ushio-Fukai, 2013). Jang and Sharkis (2007) found that low ROS levels retained the long-term self-renewal ability of hematopoietic stem cells (HSCs), and increasing ROS at appropriate levels contribute to the proliferation and migration of HSCs (Wang et al., 2009). By contrast, an excess amount of ROS limit the lifespan and self-renewing capacity of HSCs, resulting in premature senescence phenotype or apoptosis (Ito et al., 2006). These results demonstrate that the roles of ROS in regulating stem cell fate are crucial and complex. Recently, ROS have emerged as an important regulator of the germline stem cell (GSC) in *Caenorhabditis elegans* and *Drosophila melanogaster* (Senos and Jones, 2021).

Hypoxia-inducible factor 1α (HIF1α) is reported to act as a major effector of cellular redox levels and the ROS signaling via HIF1α is a key process that is critical in cell proliferation (Wu et al., 2021). HIF1α contributes to the maintenance of an undifferentiated state of various types of adult stem cells and influences their proliferation (Mohyeldin et al., 2010; Suda et al., 2011; Shyh-Chang et al., 2013). In lower invertebrates (e.g., *C. elegans* and *Drosophila*), HIF1α exhibits an important role in promoting cell proliferation and survival (Frei and Edgar, 2004; Lee et al., 2010).

In this study, we provide the information to better understand the impacts of ROS on *E. multilocularis* at the individual animal, cellular and molecular levels. We observed an obvious accumulation of ROS around the liver lesion in AE-infected mice and scavenging ROS resulted in a decreased parasite load and an impaired proliferation of the germinative cells. Increased ROS levels facilitated the growth of *E. multilocularis* and the ROS-induced activation of EmHIF1α is involved in regulating germinative cell proliferation. In summary, in addition to their damaging effects on the parasite, our results reveal an important and conducive role of ROS in the growth of *E. multilocularis* larvae during AE progression, suggesting ROS-EmERK-EmHIF1α axis as druggable targets for the development of chemotherapeutics against AE.

## Materials and methods

### Ethics statement

All animal experiments were conducted in strict accordance with China regulations on the protection of experimental animals (Regulations for the Administration of Affairs Concerning Experimental Animals, version from July 18, 2013) and specifically approved by the Institutional Animal Care and Use Committee of Xiamen University, China (Permit Number: 2013–0053).

### Cell culture and reagents

Tumor cell line HeLa was obtained from the Han’s Lab, Xiamen University (Xiamen, China). HEK-293 T cell was conserved by the State Key Laboratory of Cellular Stress Biology, Xiamen University, China. They were incubated in DMEM (Dulbecco’ s modified Eagle’ s medium; HyClone, United States) with 10% fetal bovine serum (HyClone, United States) at 37°C, 5% CO_2_. Hydrogen peroxide was obtained from Sinopharm (China). NAC was obtained from Beyotime (China). Cobalt chloride was obtained from Sigma (United States). U0126 was obtained from Selleck Chemicals (United States). YC-1 and Trolox were obtained from MedChemExpress (United States).

### Parasite *in vitro* culture and growth assay

The parasite isolate used in this study was obtained from Hulunbeier Pasture of Inner Mongolia of China and maintained by *in vivo* propagation of the parasite material in mice [supplied by Xiamen University Laboratory Animals Center, (XMULAC), China] (Tang et al., 2004). *In vitro* cultivation of metacestode vesicles was performed using HeLa conditioned medium according to a previously established protocol (Spiliotis and Brehm, 2009). Normoxia or hypoxia conditions were maintained at 37°C in the incubator with 20% O_2_ and 5% CO_2_ or 94% N_2_, 5% CO_2_, and 1% O_2_. Unless otherwise described in the text, all experiments of vesicle were performed after 48 h incubation with HeLa medium (HM). For the growth assay, vesicles (diameter ≤ 1 mm) were manually picked up and cultured with HM including beta mercaptoethanol (β-Me) in 6-well cell plates supplemented with different reagent. Parasite growth was determined by the measurement of vesicle’s diameter every 7 days. Each group contains at least 2 replicates and more than 80 vesicles in total for each group were analyzed. Two-three independent experiments were performed.

### 5-ethylnyl-20-deoxyuridine (EdU) labeling

Unless otherwise described in the text, metacestode vesicles and protoscoleces were incubated with 50 μM of EdU for 4 h and wholemount prepared according to Cheng and colleagues (Cheng et al., 2015). Click-iT-EdU Alexa Fluor 555 Imaging Kit (Life Technologies, Shanghai, China) or BeyoClick™ EdU Cell Proliferation Kit with Alexa Fluor 488 (Beyotime, Shanghai, China) was used for the detection of EdU. DNA was counterstained with 4′, 6-diamidino-2-phenylindole (DAPI) (Sigma, United States) for all labeling experiments. For the quantification of EdU^+^ cells in metacestode vesicles, 3–5 random microscopic fields per vesicle from 6 to 10 vesicles were captured and the positive cells were manually counted. For the quantification of EdU^+^ cells in protoscoleces, the protoscoleces were photographed and the image with the largest number of EdU^+^ cells was taken for counting. At least 2 labeling experiments were performed and analyzed for each control and treatment group.

### Histological analysis

For the frozen sections, liver tissue with lesions were excised form infected mice and enbedded in OCT (Optimal Cutting Temperature, Sakara, United States). Serial sections were performed with Leica cryostat (Leica Biosystems, Germany) and mounted onto slides. ROS were determined by the BBoxiProbe™ Frozen Section ROS Detection Kit (BestBio, China). EdU were determined by the BeyoClick™ EdU Cell Proliferation Kit with Alexa Fluor 594 (Beyotime, China). After treatment, total parasite lesions were excised and weighed.

There were five mice per group in lesion weight experiments, and one animal was excluded with no detectable lesion. For EdU experiment, there were five mice in the NAC group and three mice in the saline group. For the quantification of EdU^+^ cells in mice, 3–5 microscopic fields per mouse were captured and the positive cells were counted. Kunming female mice aged 8 ~ 10 weeks were used for all animal experiments, and animal procedures were approved in advance by the Institutional Animal Care and Use Committee of Xiamen University.

### Primary cell isolation and flow cytometry

After cultivation with HeLa cells for 2 months, metacestode vesicles (2 mm < diameter < 4 mm) were picked up and cultured in HM for 2 days. Metacestode vesicles were then subjected to various treatments mentioned in the text. For primary cell isolation, we referred to Spiliotis et al. (2008) with some modifications. In brief, metacestode vesicles were sheared by pipetting with a 5 mL syringe. After centrifugation (2,000 g, 5 min, room temperature) and three washing steps with PBS (15 mM NaH_2_PO_4_, 100 mM NaCl, 85 mM Na_2_HPO_4_, pH 7.4), 8 vol. of pre-warmed (37°C) trypsin (GIBCO, United States) was added to the tube. After incubation (37°C, 10 min) and adding the same volume of HM to terminate the dissociation. Then the cells were passed through a 30 μm sieve (Miltenyi Biotec, Germany) and centrifuged for 10 min at 1,000 g. The sediment was resuspended in HM. Primary cells were directly used for flow cytometry. Before the instrument analysis, primary cells were incubated with dyes 2,7-Dichlorodihydrofluorescein diacetate (DCFH-DA), DOJINDO, Japan; Hoechst 33342, Beyotime, China. The analytical and sorting instruments were Fortessa and FACSAria III (BD, United States). The data was analyzed using FlowJo X10.0 software.

### Identification and cloning of HIF1α gene of *E. multilocularis*

Published sequences of HIF1α of the human, mouse, *Drosophila*, *Xenopus*, zebrafish and *C. elegans* (Supplementary Table S1) were used as queries to BLAST the *E. multilocularis* genome database (Tsai et al., 2013) available at Wormbase database.^1^ EmuJ_000599400 was identified as the homologs HIF1α and its full coding sequences were amplified from the cDNA preparations as described previously (Brehm et al., 2000). RACE was performed using the SMART RACE cDNA Amplification Kit (Clontech, United States) according to the manufacturer’s instructions. Specific primers were used as shown in Supplementary Table S2. The domains of PAS and PAC were determined using the SMART.^2^ The phylogenetic tree was generated by the maximum likelihood method (bootstrap = 1,000) using the MEGA 7.0.26. The analysis of three-dimensional structure was generated using the SWISS-MODEL.^3^ Primers for amplification of the full coding sequences of EmHIF1α were used as shown in Supplementary Table S2.

### Co-Immunoprecipitation and western blot

EmHIF1α, EmHIF1β and HsHIF1β, tagged at their N-terminus with FLAG-tag, MYC-tag or HA-tag, respectively, were sub-cloned into pcDNA3.3 plasmid (gifts from Prof. Lin Shengcai, Xiamen University, China). The expression plasmids were co-transfected into the HEK-293 T cells with the aid of Lipofectamine™ 3,000 and Opti-MEM™ I Reduced Serum Medium (Thermo Scientific, United States). Cell lysates were harvested at 36 h post-transfection using RIPA lysis buffer (Beyotime, China). Co-Immunoprecipitation experiments were performed using anti-FLAG (Sigma, United States, RRID: AB_262044), anti-MYC (Cell Signaling Technology, United States, RRID: AB_490778) or anti-HA antibodies (Cell Signaling Technology, United States, RRID: AB_1549585) conjugated Sepharose Beads (Cell Signaling Technology, United States). Lysates of experiments were electrophoresed on SDS-polyacrylamide gels and transferred onto the PVDF membranes in a humid environment. Membranes were blocked with 5% BSA in TBST and incubated with primary antibodies at 4°C overnight. Primary antibodies: β-Tubulin (Cell Signaling Technology, United States, RRID: AB_2210545), GAPDH (Proteintech, United States, RRID: AB_2107436), Phospho-ERK (Thr185, Tyr187) (Thermo Scientific, United States, RRID: AB_2533719). Then the membranes were washed three times by TBST and incubated with the horseradish peroxidase (Invitrogen, United States) which was conjugated with the anti-rabbit or anti-mouse IgG (Thermo Scientific, United States). Blots were developed using Bio-Rad ChemiDoc Touch (Bio-Rad, United States) and analyzed by Image Lab Software.

### EmHIF1α polyclonal antibody preparation

The EmHIF1α polyclonal antibody was prepared by immunizing New Zealand Rabbit with the synthetic peptide “CDVKQFQVDSIETSN” of EmHIF1α (Genscript, United States). Purification of the anti-EmHIF1α antibody from the antiserum was performed further by protein A and peptide affinity chromatography. Western blot was performed using the EmHIF1α polyclonal antibody with a dilution of 1:1,000.

### siRNA preparation and delivery to *E. multilocularis* protoscoleces

The EmHIF1α target sequences were determined using the BLOCK-iT RNAi Designer software.^4^ The three selected siRNAs (7,013, 8,053 and 9,063) were synthesized by Biotechnology Co., Ltd. (Ribo, China). To determine the transfection efficiency, another negative control (siNC) and the FAM fluorescence label was purchased from Biotechnology Co., Ltd. (Ribo, China). The sequences of the three siRNAs targeting EmHIF1α were listed in Supplementary Table S2.

We established four siRNA groups: a negative control siRNA group (siNC) and three EmHIF1α siRNA-treated groups (7,013, 8,053 and 9,063). Electroporation was used to deliver siRNA into protoscoleces *in vitro* according to a previously established protocol (Spiliotis et al., 2010). In brief, 2000 protoscoleces were washed three times with RNAi electroporation buffer (120 mM trehalose, 20 mM HEPES, 1 mM myo-inositol, 1 mM KCl, 1 mM MgCl_2_, 1 mM K_2_HPO_4_, 0.4 mM KH_2_PO_4_ and 1 mM gluthatione, pH 6.9) and then resuspended in 100 μL electroporation buffer containing FAM-labelled control siRNA to a final concentration of 3 μM in a 1-mm electroporation cuvette. Electroporation was performed pulses once with 125 V for 20 ms by Gene Pulser II (Bio-Rad, United States). After incubation at 37°C for 10 min, 2 mL culture medium was added, and the protoscoleces were transferred to 12-well plates for an additional 60 h of incubation at 37°C in 5% CO_2_ in the dark. Then, some protoscoleces were used to collect protein samples. Remaining protoscoleces were subjected to EdU staining after incubating with 50 μM of EdU for 8 h and observed under a fluorescence microscope (SONY, Japan).

### Real-time quantitative PCR

Total vesicle RNA was collected by RNeasy Mini Kit (QIAGEN, Germany). RNA was converted to cDNA by PrimeScript RT reagent Kit with gDNA Eraser (Takara, Japan). Real-time PCR was carried out using Hieff qPCR SYBR Green Master Mix (YEASEN, China) in LightCycler 96 (Roche, Germany). *Elp* was internal control (Brehm et al., 2003). QPCR was performed with using the oligonucleotides as listed in Supplementary Table S2.

### Data analysis and statistics

Data are shown as mean ± *SD* as indicated in the respective figure legend unless otherwise indicated. The mean values of the data from the experimental groups were compared by performing a two-tailed Student’s *t*-test. SPSS 18.0 was used for statistical analysis and significance was set at *p* < 0.05. *p*-values were defined as follows: * *p* < 0.05, ** *p* < 0.01, *** *p* < 0.001, NS not significant. Asterisks without horizontal lines represent significant differences compared to the control group. Horizontal lines with asterisk on top indicate significant differences between groups.

## Results

### ROS are required for the growth of *E. multilocularis*

**Figure 1:**
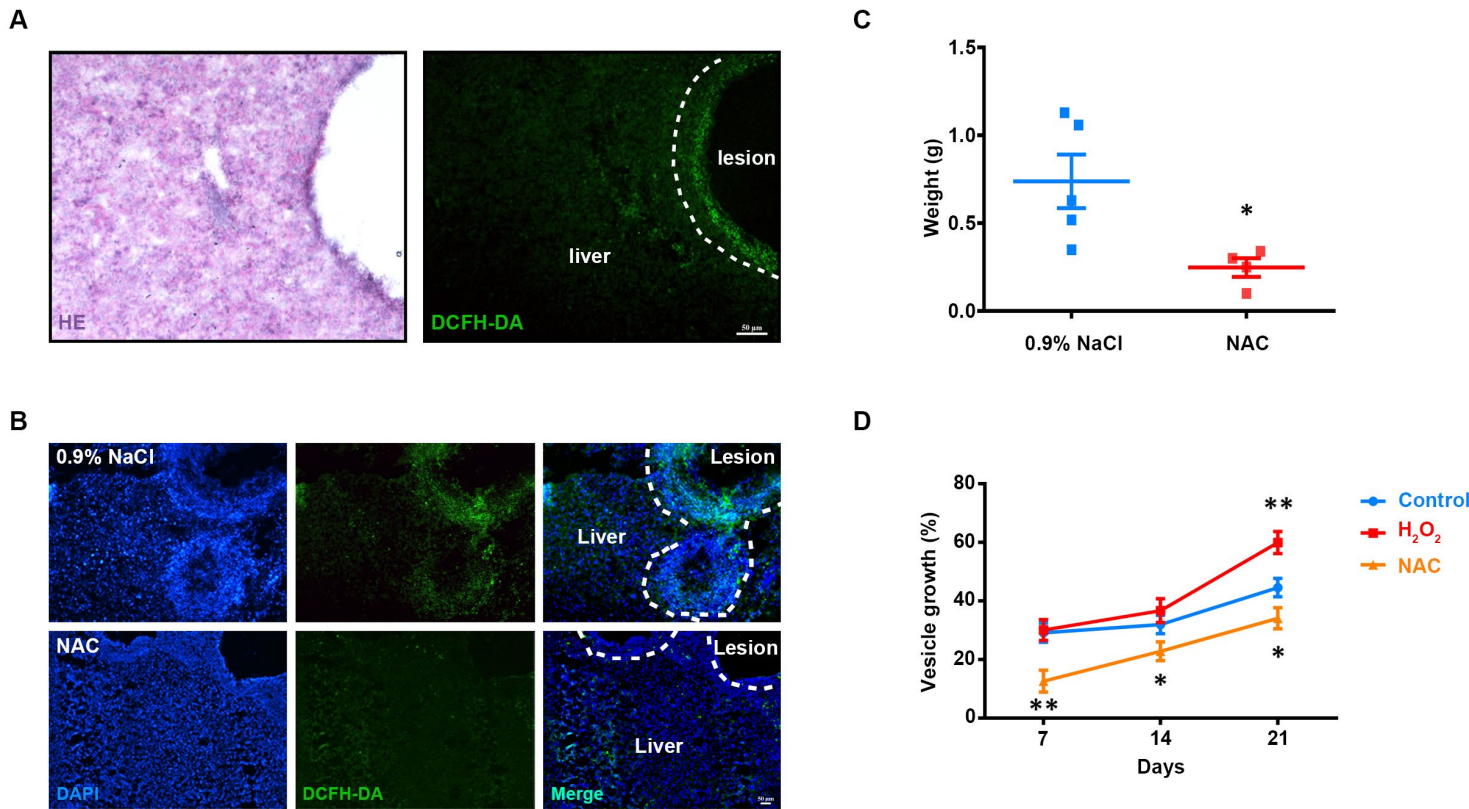
Involvement of ROS in *E. multilocularis* larval growth. **(A)** Representative hematoxylin–eosin (HE) staining and DCFH-DA staining of frozen liver sections from AE mice. Scale Bar: 50 μm. **(B,C)** Intraperitoneal injection of NAC (150 mg/kg) was commenced 2 months after infection and continued once a day for 30 days (*n* = 5). **(B)** Representative DCFH-DA staining images of frozen liver sections from AE mice and counter stained with DAPI. **(C)** Quantification of lesion weight after treatment. Each square represented a single mouse. One animal in NAC group was excluded with no detectable lesion. **(D)** Metacestode vesicles were cultivated with 1 mM NAC or 60 μM H_2_O_2_. Vesicle growth is shown as the increase of vesicle diameter as compared to day 0. Data in **(C)** was shown as mean ± *SEM*. Data in **(D)** was shown as mean ± *SD*. The significance was determined by student’ s *t*-test. ^*^p < 0.05, ^**^p < 0.01.

Infection with parasites induces host immune responses to produce ROS. We first examined the *in vivo* ROS pattern of AE in the mouse liver using the ROS-sensitive dye DCFH-DA (2,7-Dichlorodihydrofluorescein diacetate) and found an obvious ROS accumulation around the lesion (Figure 1A). To investigate the effect of ROS on the growth of metacestode larvae, we then injected ROS scavenger NAC (N-Acetyl-L-Cysteine) into the mice and found that the accumulation of ROS was significantly reduced (Figure 1B). Interestingly, the parasite weight was decreased after NAC treatment (Figure 1C). To confirm this effect of ROS on parasite growth *in vitro*, we added NAC to the culture medium and found that the growth rate of the metacestode vesicles greatly decreased. By contrast, addition of H_2_O_2_ (hydrogen peroxide) increased the growth rate (Figure 1D). These results suggest that the growth of *E. multilocularis* larvae requires the participation of ROS.

### ROS are involved in germinative cell proliferation

**Figure 2:**
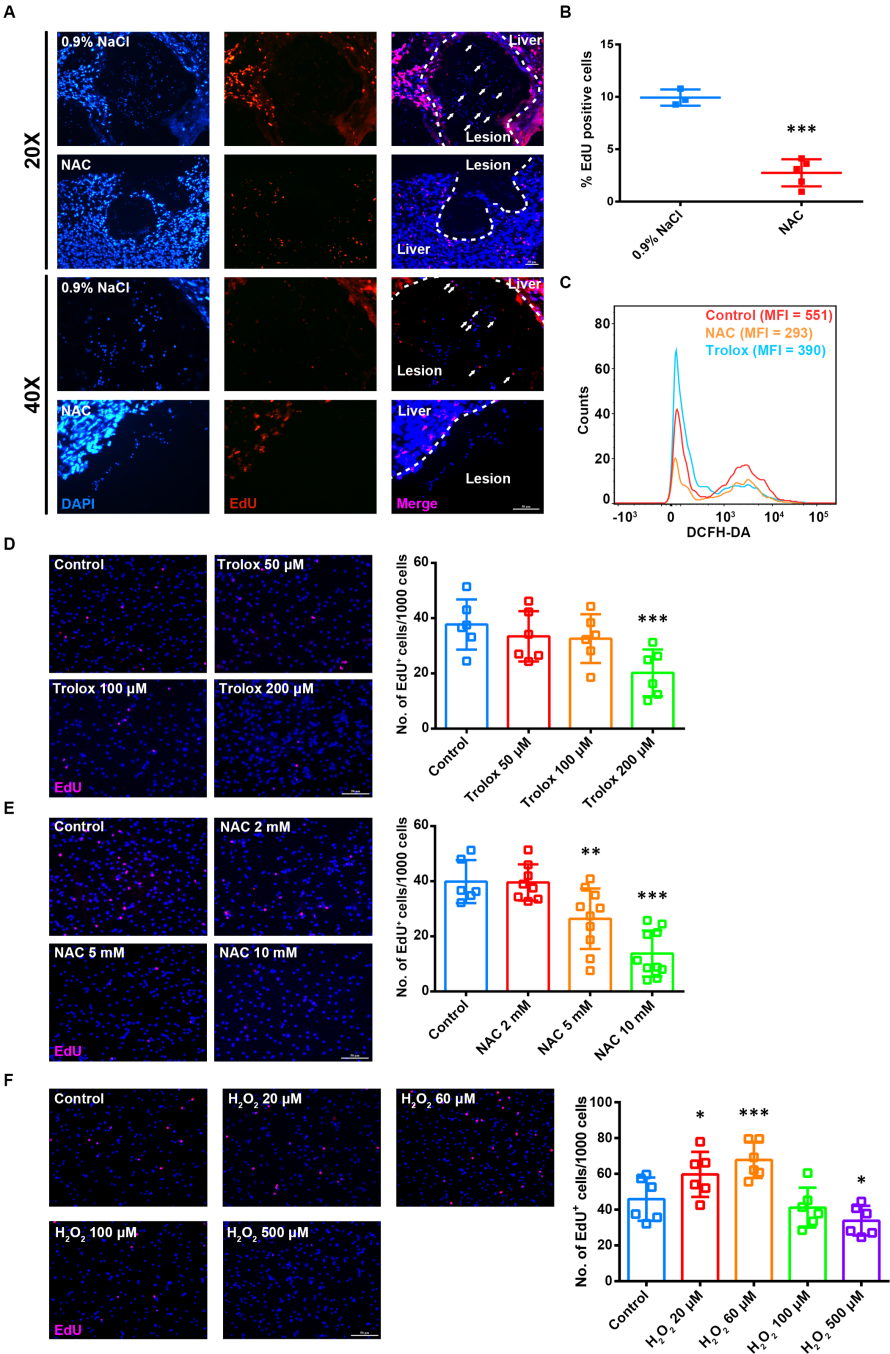
ROS levels are associated with proliferation of germinative cells. **(A)** Intraperitoneal injection of NAC (150 mg/kg) was commenced 3 months after infection with metacestode vesicles and continued once a day for 14 days. Mice were given intraperitoneal injection of EdU (10 mg/kg) 7 consecutive days before sample collection (once a day). Representative EdU staining images of frozen liver sections from AE mice. Scale Bar: 50 μm. White arrows indicated EdU^+^ cells in lesions. **(B)** Quantification of the percentage of EdU^+^ cells was shown. Each square represented a single mouse. **(C)** Flow cytometric analysis of ROS level in the vesicle primary cells treated with 5 mM NAC or 200 μM Trolox for 8 h. MFI is mean fluorescence intensity. **(D–F)** Metacestode vesicles were treated with 50–200 μM Trolox **(D)** or 2–10 mM NAC **(E)** or 20–500 μM H_2_O_2_ **(F)** for 24 h and then stained for EdU. Scale Bar: 50 μm. Quantifications of EdU^+^ cells were shown on the right for each panel. Each square represented a single vesicle. Data in **(B,D–F)** were shown as mean ± *SD*. The significance was determined by student’ s *t*-test. ^*^p < 0.05, ^**^p < 0.01, ^***^p < 0.001.

The proliferation of germinative cells on the germinal layer is the basis of *E. multilocularis* larval growth (Koziol et al., 2014). So we examined the effect of ROS on the proliferation of germinative cells *in vivo*. AE-infected mice were administered to NAC treatment first, followed by intraperitoneal injection of EdU for labeling the proliferating cells. The results showed that the number of EdU^+^ cells in metacestode decreased greatly in the NAC treatment group (Figures 2A,B). Then we treated *in vitro-*cultured metacestode vesicles with two ROS scavengers NAC and Trolox (6-hydroxy-2,5,7,8-tetramethylchroman-2-carboxylic acid) respectively, both of which could efficiently down-regulate the ROS levels in the primary cells of metacestode vesicles (Figure 2C). We found that the number of EdU^+^ cells decreased after NAC/Trolox treatment in a dose-dependent manner (Figures 2D,E), accompanied with a significant down-regulation of the mRNA levels of cell cycle-related genes (Supplementary Figures S1A,B). Furthermore, we treated the metacestode vesicles with H_2_O_2_. The results showed that the number of EdU^+^ cells and the mRNA levels of cell cycle-related genes were increased greatly after 60 μM H_2_O_2_ treatment, whereas 500 μM H_2_O_2_ reduced the number of EdU^+^ cells (Figure 2F and Supplementary Figure S1C). These results suggest that the moderate ROS can promote the proliferation of germinative cells and too low or too high ROS levels are not conducive to proliferation.

### ROS-EmHIF1α axis is involved in regulating the proliferation of germinative cells

HIF1α (hypoxia inducible factor 1α) is reported to act as a major effector of cellular redox levels (Lee et al., 2017). We excavated the genome information of *E. multilocularis* by BLAST analyses using human, *D. melanogaster*, and *C. elegans* HIF1α as the queries and procured as best hit protein encoded by locus EmuJ_000599400 (Supplementary Figures S2, S3). *E. multilocularis* HIF1α homolog (EmHIF1α) contains conserved HIF-characterized PAS and PAC domains (Supplementary Figure S2A), has a close evolutionary relationship with *C. elegans* HIF1α and exhibits a three-dimensional structure similar to human HIF1α (Supplementary Figures S2B,C). In mammals, HIF1α is stabilized, accumulated, and forms heterodimers with HIF1β to transcriptionally activate various downstream genes (Nandal et al., 2011). We also identified a HIF1β homologue of *E. multilocularis* by genome mining (EmuJ_000805200) (Supplementary Figures S2D–F). Co-IP from HEK-293 T cell line indicated that EmHIF1α immunoprecipitated with human HIF1β and EmHIF1β (Supplementary Figures S2G,H), suggesting a conserved complexing mechanism of HIF1 subunits in *E. multilocularis*. We then generated a polyclonal antibody against EmHIF1α, which effectively detected the recombinant His-tagged EmHIF1α protein as well as the endogenous EmHIF1α in the *in vitro*-cultivated metacestode vesicles (Supplementary Figures S2I,J). Further, we examined the expression of EmHIF1α in metacestode vesicles and found that EmHIF1α expression was increased under hypoxia (Supplementary Figure S2K).

**Figure 3:**
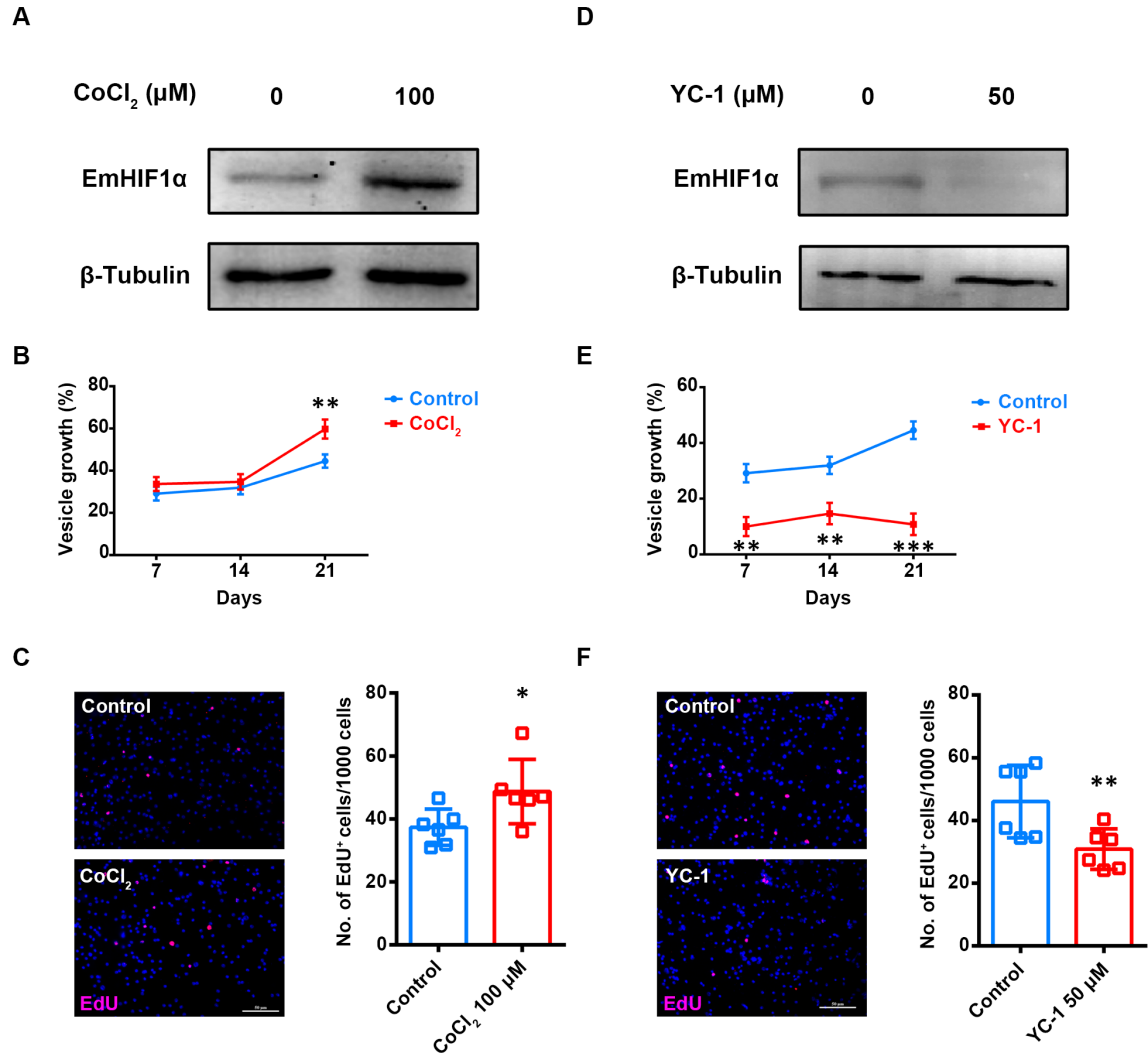
EmHIF1α participates in regulating the proliferation of germinative cells. **(A,D)** Representative western blot for EmHIF1α expression from metacestode vesicles treated by 100 μM CoCl_2_ **(A)** or 50 μM YC-1 **(D)** for 8 h. **(B,E)** Metacestode vesicles were cultivated with 50 μM CoCl_2_ **(B)** or 50 μM YC-1 **(E)**. Vesicle growth is shown as the increase of vesicle diameter as compared to day 0. **(C,F)** Metacestode vesicles treated with 100 μM CoCl_2_ **(C)** or 50 μM YC-1 **(F)** for 24 h were stained for EdU. Scale Bar: 50 μm. Quantifications of EdU^+^ cells were shown in the right panel. Each square represented a single vesicle. Data in **(B,C,E,F)** were shown as mean ± *SD*. The significance was determined by student’ s *t*-test. ^*^p < 0.05, ^**^p < 0.01, ^***^p < 0.001.

To investigate the role of EmHIF1α in germinative cells proliferation, we treated metacestode vesicles with CoCl_2_ (cobaltous chloride), an eminent hypoxia mimetic chemical and inducer of HIF1α (Munoz-Sanchez and Chanez-Cardenas, 2019). The results showed that CoCl_2_ treatment resulted in a great induction of EmHIF1α (Figure 3A). Along with this, the growth rate of metacestode vesicles and the number of EdU^+^ cells significantly increased (Figures 3B,C) and the mRNA levels of cell cycle-related genes were up-regulated (Supplementary Figure S4A). We found that YC-1 [3-(5′-hydroxymethyl-2′-furyl)-1-benzyl indazole], an inhibitor of HIF1α (Kim et al., 2006), could reduce the expression of EmHIF1α (Figure 3D). After the treatment with YC-1, the growth rate of metacestode vesicles, the number of EdU^+^ cells and the mRNA levels of proliferation marker genes were decreased significantly (Figures 3E,F and Supplementary Figure S4B). These results suggest that EmHIF1α is involved in the proliferation of germinative cells.

**Figure 4:**
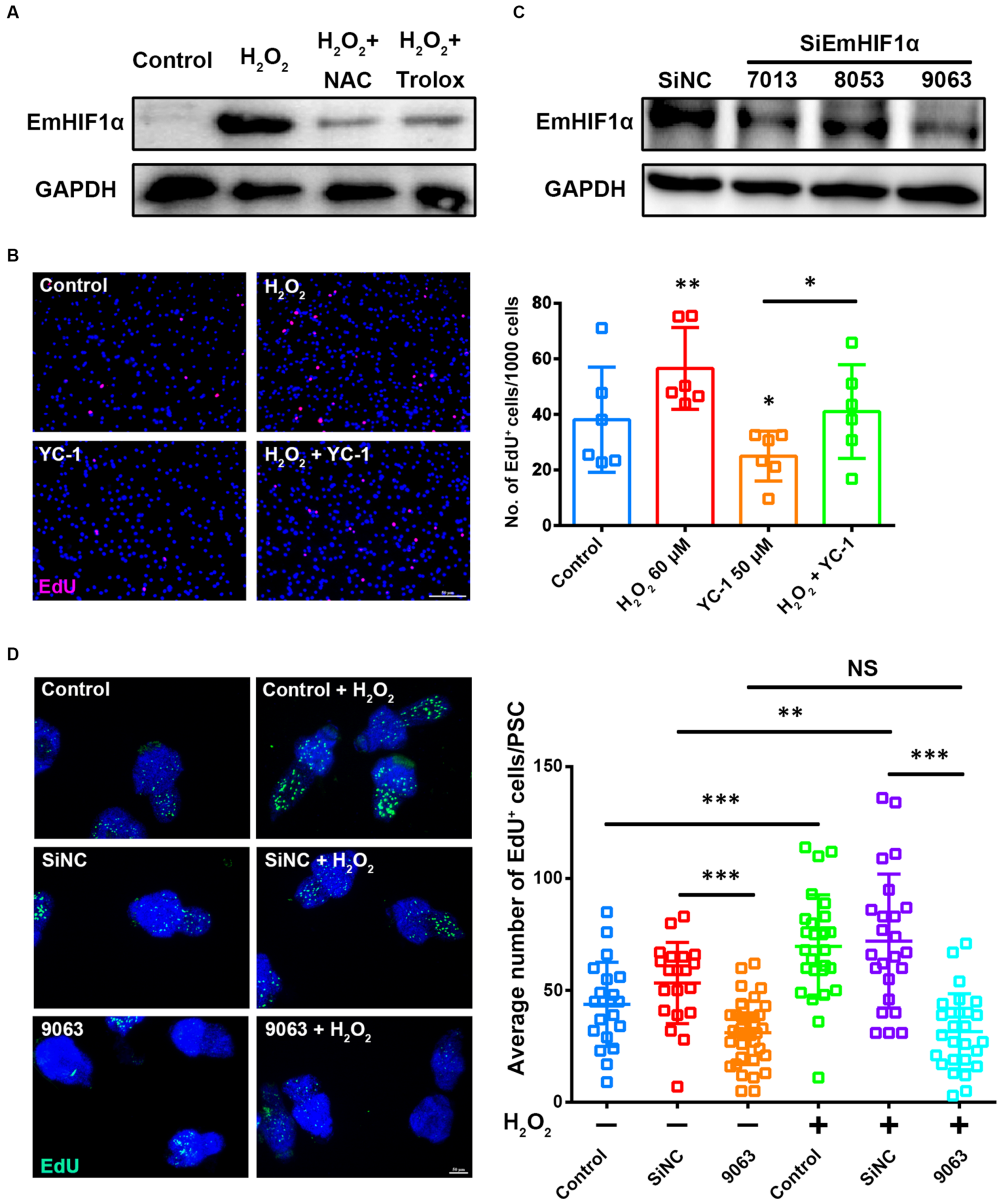
The ROS-EmHIF1α axis regulates germinative cell proliferation. **(A)** Western blot for EmHIF1α expression from the metacestode vesicles treated with 60 μM H_2_O_2_, 5 mM NAC or 200 μM Trolox for 8 h. **(B)** Metacestode vesicles treated with 60 μM H_2_O_2_ or 50 μM YC-1 for 24 h were stained for EdU. Scale Bar: 50 μm. Quantification of EdU^+^ cells was shown on the right. Each square represented a single vesicle. **(C)** Representative western blot showing EmHIF1α protein levels for siNC and different siEmHIF1α sequences (7,013, 8,053 and 9,063). **(D)** Protoscoleces were treated with 50 μM EdU and 60 μM H_2_O_2_ for 8 h after 60 h of the siRNA transfer. Slides were sealed for photographs and quantitative analysis after EdU color development. Scale Bar: 50 μm. Quantification of EdU^+^ cells was shown in the right panel. Each square represented a single protoscolex (*n* ≥ 20). Data in **(B,D)** were shown as mean ± *SD*. The significance was determined by student’ s *t*-test. NS *p* > 0.05, ^*^p < 0.05, ^**^p < 0.01, ^***^p < 0.001.

We found that increasing the ROS levels in metacestode vesicles significantly enhanced EmHIF1α expression and this phenotype could be largely restored by NAC or Trolox (Figure 4A). To determine whether ROS promote the proliferation of germinative cells by regulating EmHIF1α, the metacestode vesicles were treated with YC-1 and/or H_2_O_2_. The results showed that YC-1 significantly compromised the H_2_O_2_-induced proliferation of germinative cells (Figure 4B). We further alternatively performed siRNA to suppress EmHIF1α expression in protoscoleces. The results showed that the number of EdU^+^ cells was significantly decreased by EmHIF1α knockdown in comparison with control siRNA (siNC). In addition, the increased number of EdU^+^ cells induced by H_2_O_2_ was clearly inhibited by EmHIF1α knockdown (Figures 4C,D). These results suggest that the ROS-EmHIF1α axis may be important in the growth of metacestode larvae and the proliferation of germinative cells.

### EmERK regulates EmHIF1α-mediated germinative cell proliferation

**Figure 5:**
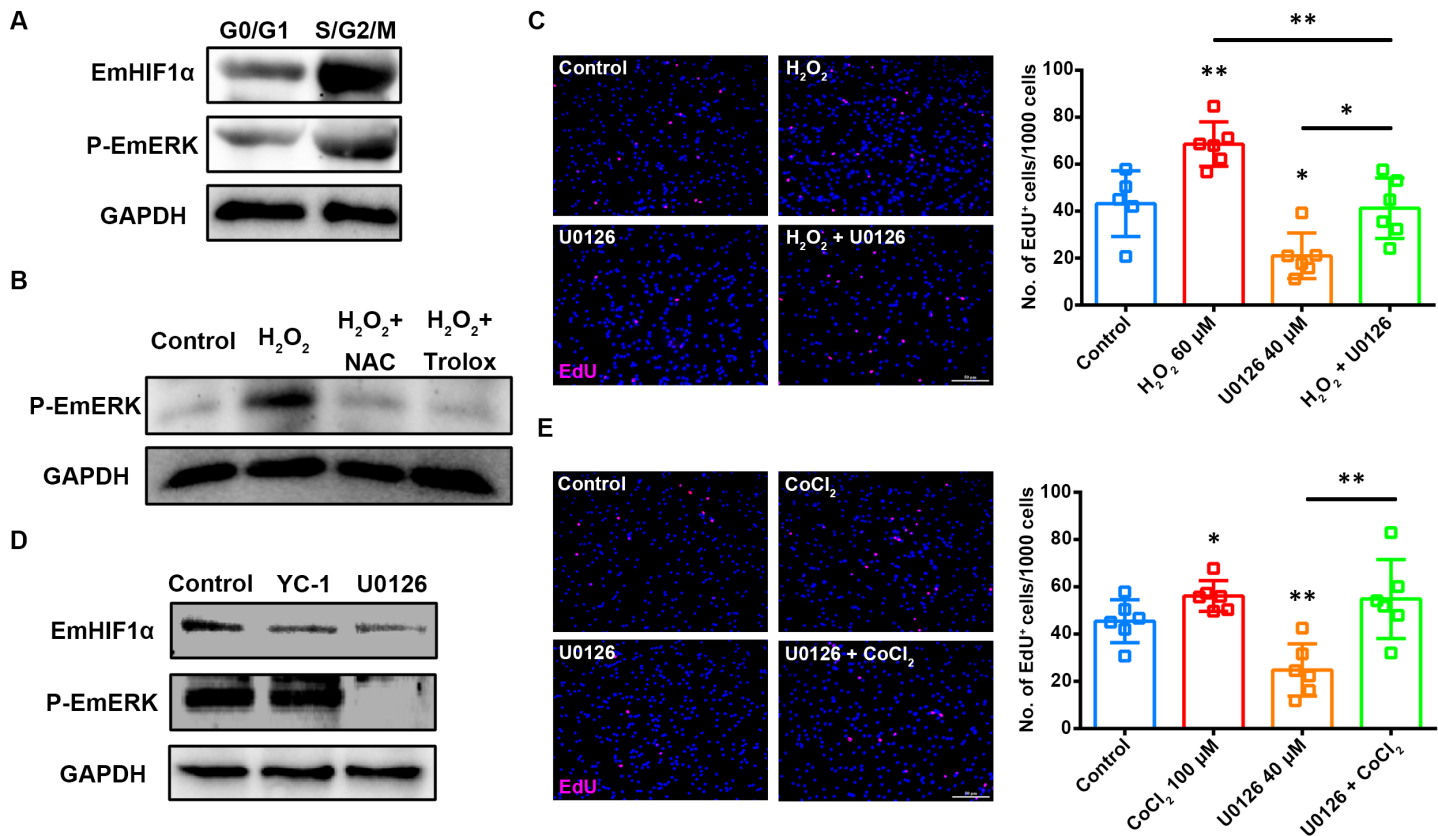
EmERK is involved in ROS-EmHIF1α axis-regulated germinative cell proliferation. **(A)** Cells from freshly isolated metacestode vesicles primary cells were stained with Hoechst 33342 for sorting based on DNA content. The expressions of EmHIF1α and p-EmERK (Thr185/Tyr187) in the G0/G1 and S/G2/M cells were analyzed by western blot. **(B)** Western blot for p-EmERK expression in the metacestode vesicles treated with 60 μM H_2_O_2_, 5 mM NAC or 200 μM Trolox for 8 h. **(C,E)** Metacestode vesicles treated with 60 μM H_2_O_2_ and 40 μM U0126 **(C)** or 100 μM CoCl_2_ and 40 μM U0126 **(E)** for 24 h were stained for EdU. Scale Bar: 50 μm. Quantifications of EdU^+^ cells were shown on the right, respectively. Each square represented a single vesicle. **(D)** Western blot for EmHIF1α and p-EmERK expression in the metacestode vesicles treated with 50 μM YC-1 or 40 μM U0126 for 8 h. Data in **(C,E)** were shown as mean ± *SD*. The significance was determined by student’ s *t*-test. ^*^p < 0.05, ^**^p < 0.01.

ROS have been reported to induce HIF1α protein synthesis through the ERK signaling transduction pathway (Mottet et al., 2002). The previous studies have shown that the activation of EmERK signaling promotes the proliferation of germinative cells in *E. multilocularis* (Spiliotis et al., 2005, 2006; Gelmedin et al., 2010; Cheng et al., 2017). To know whether EmERK regulates the proliferation of germinative cells by involving ROS-EmHIF1α axis, we first isolated primary cells from the *in vitro-*cultivated metacestode vesicles (Supplementary Figures S5A,B) and analyzed the expression of phosphorylated EmERK (p-EmERK) and EmHIF1α in the sorted S/G2/M and G0/G1 cells. The results showed that p-EmERK and EmHIF1α were highly expressed in the S/G2/M cells (Figure 5A), suggesting that they may function in regulating germinative cell proliferation. Then we found that, similar to EmHIF1α, EmERK phosphorylation was also regulated by ROS (Figure 5B). We next treated the metacestode vesicles with the MEK/ERK inhibitor U0126 (Cheng et al., 2020) and found that U0126 significantly compromised the H_2_O_2_-induced proliferation of germinative cells (Figure 5C). In order to explore the effect of p-EmERK on EmHIF1α expression, we inhibited EmERK phosphorylation and EmHIF1α expression respectively, and found that the protein level of p-EmERK did not change significantly upon YC-1 treatment, while the treatment with U0126 effectively blocked the expression of EmHIF1α (Figure 5D). Furthermore, we activated EmHIF1α while inhibiting p-EmERK and found that the proliferation of germinative cells was restored (Figure 5E). Taken together, these results suggest that ROS stabilize the expression of EmHIF1α by phosphorylating EmERK, which ultimately affects the proliferation of germinative cells.

## Discussion

There is increasing evidence to indicate that ROS have an important role in regulating the homeostasis of proliferating cells. Moderate levels of ROS are required for normal proliferating cell function in many tissues. Jones et al. (2013) have elucidated that oxidants have a positive role in terms of intestinal stem cell homeostasis. Similarly, ROS are shown to promote neural stem cell self-renewal (Le Belle et al., 2011). A similar observation has also been recently made in spermatogonial stem cells (Morimoto et al., 2021). In addition to normal proliferating cells, a modest increase of ROS contributes to cancer stem cells (CSC) proliferation, whereas excessive levels of ROS induce CSC apoptosis or necrosis (Qian et al., 2018). Our research indicates that ROS intricately modulate the proliferation of germinative cells in *E. multilocularis*. Specifically, we observed that reduced ROS levels inhibited germinative cell proliferation, while increased ROS levels enhanced it, albeit with a decline in the number of germinative cells at excessive concentrations. These findings provide novel evidence for the double-edged function of ROS regulating cell proliferation in multicellular parasitic worms, as well as a new perspective on the interaction between helminths and their hosts.

The role of ROS in triggering signaling pathways for cell proliferation has been well established. ROS have been shown to activate MAP kinases, including ERK1/2, that regulates cell proliferation and differentiation by stimulating the synthesis of HIF1α (Malekan et al., 2021; Luo et al., 2022). Our investigations into *E. multilocularis* reveal that ROS-induced phosphorylation of EmERK notably enhances germinative cell proliferation by upregulating EmHIF1α, suggesting innovative therapeutic avenues against AE by targeting the ROS/EmERK/EmHIF1α axis. Anyway, the clinical use of inhibitors targeting the signaling axis (e.g., NAC, U0126 and YC-1) for the treatment of human AE needs further investigations. Additionally, there is evidence for an important role of ROS in modulating Notch signaling, which are vital for restraining differentiation and maintaining stemness (Duncan et al., 2005; Hamanaka et al., 2013). Furthermore, ROS can serve a pro-survival role by antagonizing PTEN (Zhang et al., 2020). A major target of PTEN is protein kinase B (Akt), a central regulator of cell survival and pro-oncogenic signaling (Palma et al., 2023). Our on-hand preliminary experimental results suggest that Akt and Notch are also activated in the metacestode vesicles upon H_2_O_2_ treatment. In mammalian cells, Akt and Notch play a role in regulating HIF1α (Zhang et al., 2019; Li et al., 2020). Whether the similar mechanism exists in *E. multilocularis* should be investigated in the future.

During parasitic infections, host immune defenses may be considered one of the most sophisticated products of interspecific interactions. ROS and oxidative stress perform critical functions in protecting host against infectious agents. ROS produced by host neutrophils and macrophages cause irreversible damage to cellular structures and components that are required for parasitic viability (Sorci and Faivre, 2009). Redox-active antiparasitic drugs that either promote ROS generation or inhibit cellular antioxidant systems will lead to redox imbalance by pushing ROS levels above a certain threshold level that will ultimately lead to parasite death (Pal and Bandyopadhyay, 2012). *E. multilocularis* larvae present as a slowly and perpetually growing mass and we found an obvious ROS accumulation around the liver lesion in the infected mice, consistent with the recent report that immune cells gather around the lesion and generate an immune response in the advanced stages of AE (Autier et al., 2023). Contrary to the traditional beliefs asserting the detrimental impact of ROS on parasite growth, we found that ROS in the AE lesion microenvironment may be actively involved in regulating *E. multilocularis* larval growth. Specifically, we observed that reduced ROS levels gave rise to the decreased parasite mass and germinative cell proliferation. In addition, our *in vitro* experiments found that the effect of ROS on larval growth was achieved by regulating the proliferation of germinative cells. These results suggest that *E. multilocularis* may adapt the ROS in host microenvironment under oxidative stress and that ROS may be beneficial to cellular proliferation and larval growth. More dedicated *in vivo* experiments are need to further illustrate the detailed mechanism in the future work. Anyway, the present sutdy enriches our understanding of the adaptive strategies that multicellular parasitic worms employ against host oxidative stress.

The interaction between parasites and their hosts are highly complex, and most of the research on this interaction are conducted in unicellular parasites. *Toxoplasma* and *Theileria* lead to extensive changes in host transcriptome regulation and these changes can have drastic effects on host cell phenotypes, including stress and inflammatory responses (Cheeseman and Weitzman, 2015). *Plasmodium* and *Leishmania* establish complex membrane structures inside host cells to change phagocytosis (Fraser et al., 2023). For the multicellular parasite, the parasite–host interaction is more complex. It could be divided into two equal important aspects. One is the effects of parasite on the host. Extensive research indicates that helminths use small microRNAs to manipulate their host’s immune responses by ‘cross-kingdom’ gene regulation (Chowdhury et al., 2023). Advances in single-cell sequencing have significantly clarified the influence of the impact of *Echinococcus* on the immune microenvironment of the host (Yasen et al., 2021; Jiang T. et al., 2022; Jiang X. et al., 2022; Wu et al., 2023). The second aspect addresses the effects of host on the parasite, including adapting to host microenvironment and evading host immunity. Compared to studies on the effects of parasite on the host, few research on the effects of host on the parasite has been reported (Wendt et al., 2020). As to AE, limited research suggests that *Echinococcus*-host cross-communication via evolutionarily conserved signalling pathways. Brehm and colleagues found that host insulin is most likely governing larval development via stimulating parasite insulin signaling pathway (Hemer et al., 2014). Our previous research found that host EGF may regulate germinative cells proliferation by stimulating the EGFR signaling pathway (Cheng et al., 2017; Feng et al., 2022). In this study, our findings underscore the role of ROS-induced EmERK phosphorylation in promoting larval growth through EmHIF1α upregulation, suggesting the beneficial effect of the microenvironmental ROS on parasite. ROS contributing to parasite persistence is well established in *Trypanosoma*, which is an intracellular single-cell parasite (Paiva et al., 2012). To our knowledge, our study is the first one showing that ROS might be conducive for the growth of extracellular multicellular parasites.

In conclusion, our study advances the understanding of the integral yet overlooked role of ROS in promoting germinative cell proliferation and metacestode larval growth of *E. multilocularis*, and complements the understanding of *Echinococcus* adaptation to host oxidative stress. To ensure the long-term survival in host tissues, instead of being eliminated by host immune response, *E. multilocularis* has developed a series of adaptive mechanisms under the oxidative stress. The important manifestation of this adaptation is the strategic utilization of ROS, enhancing both germinative cell proliferation and larval growth. Elucidating these adaptive strategies may offer potential pathways for novel therapeutic interventions for AE.

## Data availability statement

The original contributions presented in the study are publicly available. This data can be found here: https://osf.io/r9wqn/.

## Ethics statement

The animal study was approved by Institutional Animal Care and Use Committee of Xiamen University, Xiamen, China. The study was conducted in accordance with the local legislation and institutional requirements.

## Author contributions

YT: Investigation, Validation, Writing – original draft, Writing – review & editing, Methodology. ZC: Conceptualization, Project administration, Writing – review & editing, Funding acquisition, Supervision. DG: Investigation, Writing – review & editing. ZX: Writing – review & editing, Methodology. HW: Writing – review & editing, Methodology. XL: Investigation, Writing – review & editing. HT: Writing – review & editing. FL: Writing – review & editing. DL: Writing – review & editing. YW: Conceptualization, Project administration, Writing – review & editing, Funding acquisition, Supervision.

## Footnotes

^1^https://parasite.wormbase.org/Echinococcus_multilocularis_prjeb122/Info/Index/

^2^http://smart.embl-heidelberg.de/

^3^https://swissmodel.expasy.org/

^4^https://rnaidesigner.invitrogen.com/rnaiexpress/

## Supplementary material

SUPPLEMENTARY FIGURE S1 ROS regulate the expression of cell cycle-related genes in metacestode vesicles. Real-time PCR analysis of the mRNA expressions of cell cycle-related factors in the metacestode vesicles treated with 5 mM NAC **(A)**, 200 μM Trolox **(B)** or 60 μM H_2_O_2_ **(C)** for 4 hours. Data were shown as mean ± *SD*. The significance was determined by student’ s *t*-test. ^*^*p* < 0.05, ^**^*p* < 0.01, ^***^*p* < 0.001.

SUPPLEMENTARY FIGURE S2 Identification of the HIF1α homolog in *E. multilocularis.* **(A)** Alignment of the PAS and PAC domains of EmHIF1α with that of other HIF1α members. Similar residues were marked black if they were present in ≥ 80 % of the sequences. **(B)** Phylogenetic analysis of EmHIF1α. The PAS and PAC domain of HIF1α members were used for phylogenetic tree construction (bootstrap=1,000). **(C)** Comparison of the predicted three-dimensional structures of the PAS and PAC domains of human HIF1α and EmHIF1α. **(D)** Alignment of the HLH and PAS domains of EmHIF1β with that of other HIF1β members. Similar residues were marked black if they were present in ≥ 100 % of the sequences. **(E)** Phylogenetic analysis of EmHIF1β. The HLH and PAS domain of HIF1β members were used for phylogenetic tree construction (bootstrap=1,000). **(F)** Comparison of the predicted three-dimensional structures of the HLH and PAS domains of human HIF1α and EmHIF1β. **(G–H)** Identification of the EmHIF1α-EmHIF1β **(G)** interaction or EmHIF1α-HIF1β **(H)** interaction in HEK-293T by Co-IP, and detected by immunoblotting. **(I)** Lysates of bacteria expressing His-tagged EmHIF1α were analyzed by western blotting using the anti-His tag antibody (left) or the anti-EmHIF1α antibody (right). Line 1 and 4: empty vector control; line 2 and 5: IPTG uninduced; line 3 and 6: induced with IPTG for 4 hours. **(J)** Total protein of *in vitro*-cultured metacestode vesicles was analyzed with the anti-EmHIF1α antibody. **(K)** Western blot analysis of EmHIF1α expression in the metacestode vesicles cultured under normoxia (20 % O_2_) or hypoxia (1 % O_2_). Em: *Echinococcus multilocularis*, Ce: *Caenorhabditis elegans*, Dm: *Drosophila melanogaster*, Dr: *Danio rerio*, Xl: *Xenopus laevis*, Hs: *Homo sapiens*, Mm: *Mus musculus*.

SUPPLEMENTARY FIGURE S3 Gene sequence of *emhif1α*. Introns were identified by comparing the genomic DNA sequence with cDNA. The length of each intron and its partial sequence including the canonical GT-AG-dinucleotides at the 5’ splice donor and the 3’ splice acceptor sites (italic lowercase) are given. The translational start codon is indicated by a single line “—”. The stop codon is indicated by a double line “”. Sequences of 3’ untranslated regions are shown in lowercase. Coding sequence is shown in uppercase. Note that the actual size (1,347 bp) of the *emhif1α* smaller than predicted (1,839 bp). The *emhif1α* gene spans a genomic region of 7,381 kb and comprised 8 exons, separated by 7 introns.

SUPPLEMENTARY FIGURE S4 EmHIF1α agonist and inhibitor regulate the expression of cell cycle-related genes in metacestode vesicles. Real-time PCR analysis of the mRNA expressions of cell cycle-related factors in the metacestode vesicles treated with 100 μM CoCl_2_ **(A)** or 50 μM YC-1 for 4 hours **(B)**. Data were shown as mean ± *SD*. The significance was determined by student’ s *t*-test. ^*^*p* < 0.05, ^**^*p* < 0.01, ^***^*p* < 0.001.

SUPPLEMENTARY FIGURE S5 Flow cytometry sorting strategy. **(A)** Gating strategy for the primary cells freshly isolated from metacestode vesicles. Upper left: chief cell population (black oval), upper right: chief cell population after removing adhesions (black rectangle), lower right: chief cell population after removing fragmented fluorescence (right line segment), lower left: DNA content of the primary cells. **(B)** Freshly isolated primary cells were stained with Hoechst 33342 for sorting based on DNA content. mRNA expressions of a set of cell cycle-related factors were analyzed after cell sorting. Data in **(B)** were shown as mean ± *SD*. The significance was determined by student’ s *t*-test. ^***^*p* < 0.001.
