# Supplementary figures and images for: ROS are required for the germinative cell proliferation and metacestode larval growth of Echinococcus multilocularis
Source: Front Microbiol. 2024 Jun 7;15:1410504. doi: 10.3389/fmicb.2024.1410504 (PMC11190091; doi:10.3389/fmicb.2024.1410504)

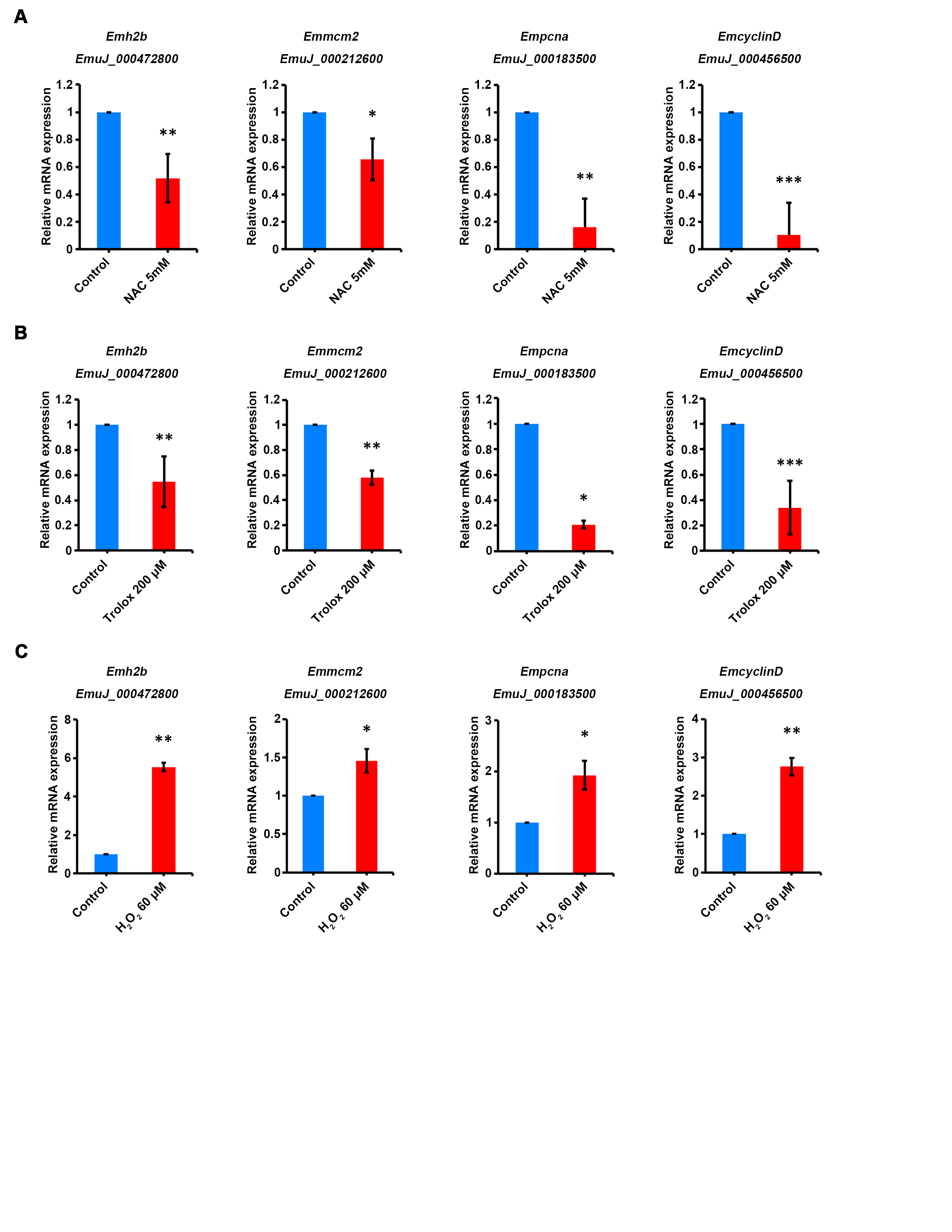

Supplement: SUPPLEMENTARY FIGURE S1 — ROS regulate the expression of cell cycle-related genes in metacestode vesicles. Real-time PCR analysis of the mRNA expressions of cell cycle-related factors in the metacestode vesicles treated with 5 mM NAC (A), 200 μM Trolox (B) or 60 μM H2O2 (C) for 4 hours. Data were shown as mean ± SD. The significance was determined by student’ s t-test. *p < 0.05, **p < 0.01, ***p < 0.001. [file Image_1.TIF]

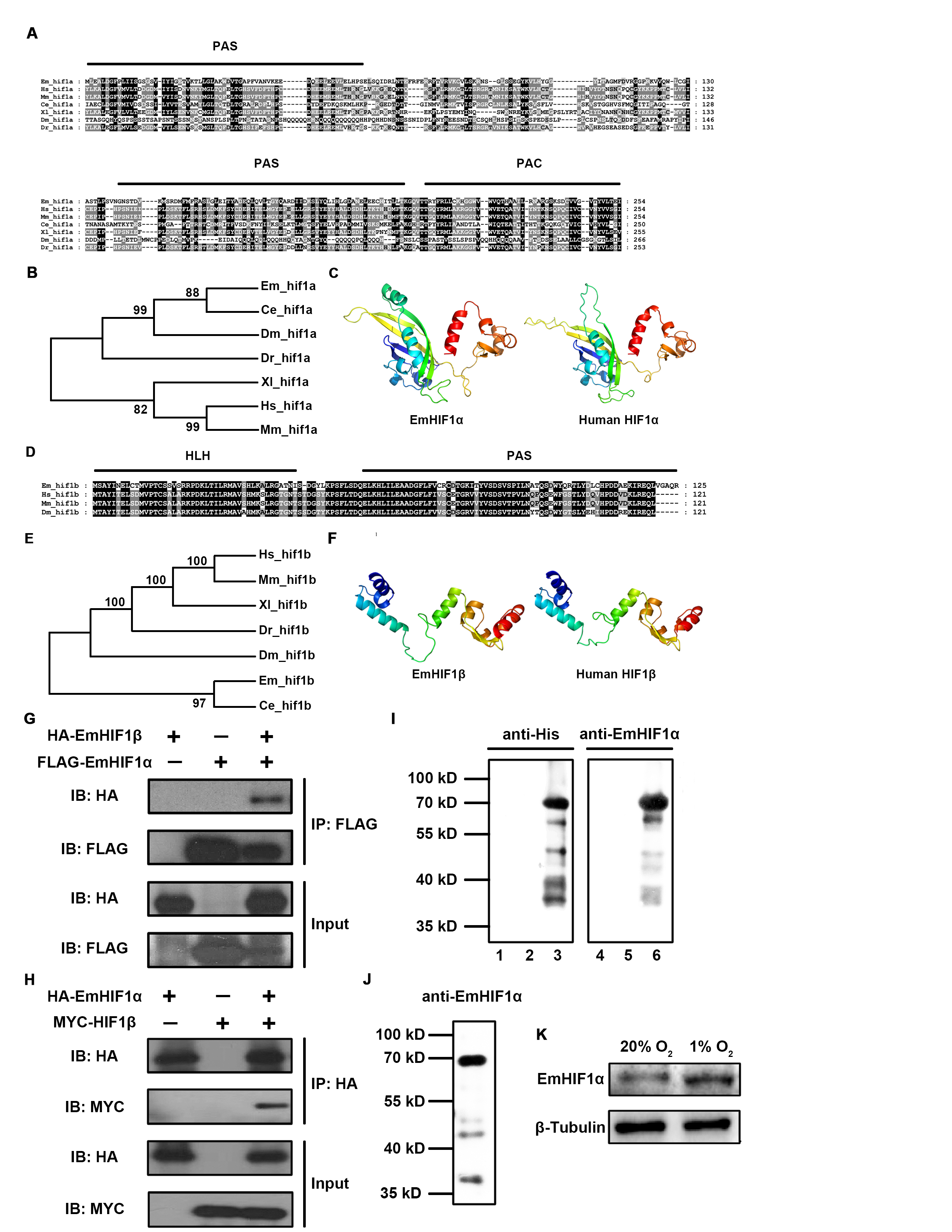

Supplement: SUPPLEMENTARY FIGURE S2 — Identification of the HIF1α homolog in E. multilocularis. (A) Alignment of the PAS and PAC domains of EmHIF1α with that of other HIF1α members. Similar residues were marked black if they were present in ≥ 80 % of the sequences. (B) Phylogenetic analysis of EmHIF1α. The PAS and PAC domain of HIF1α members were used for phylogenetic tree construction (bootstrap=1,000). (C) Comparison of the predicted three-dimensional structures of the PAS and PAC domains of human HIF1α and EmHIF1α. (D) Alignment of the HLH and PAS domains of EmHIF1β with that of other HIF1β members. Similar residues were marked black if they were present in ≥ 100 % of the sequences. (E) Phylogenetic analysis of EmHIF1β. The HLH and PAS domain of HIF1β members were used for phylogenetic tree construction (bootstrap=1,000). (F) Comparison of the predicted three-dimensional structures of the HLH and PAS domains of human HIF1α and EmHIF1β. (G–H) Identification of the EmHIF1α-EmHIF1β (G) interaction or EmHIF1α-HIF1β (H) interaction in HEK-293T by Co-IP, and detected by immunoblotting. (I) Lysates of bacteria expressing His-tagged EmHIF1α were analyzed by western blotting using the anti-His tag antibody (left) or the anti-EmHIF1α antibody (right). Line 1 and 4: empty vector control; line 2 and 5: IPTG uninduced; line 3 and 6: induced with IPTG for 4 hours. (J) Total protein of in vitro-cultured metacestode vesicles was analyzed with the anti-EmHIF1α antibody. (K) Western blot analysis of EmHIF1α expression in the metacestode vesicles cultured under normoxia (20 % O2) or hypoxia (1 % O2). Em: Echinococcus multilocularis, Ce: Caenorhabditis elegans, Dm: Drosophila melanogaster, Dr: Danio rerio, Xl: Xenopus laevis, Hs: Homo sapiens, Mm: Mus musculus. [file Image_2.TIF]

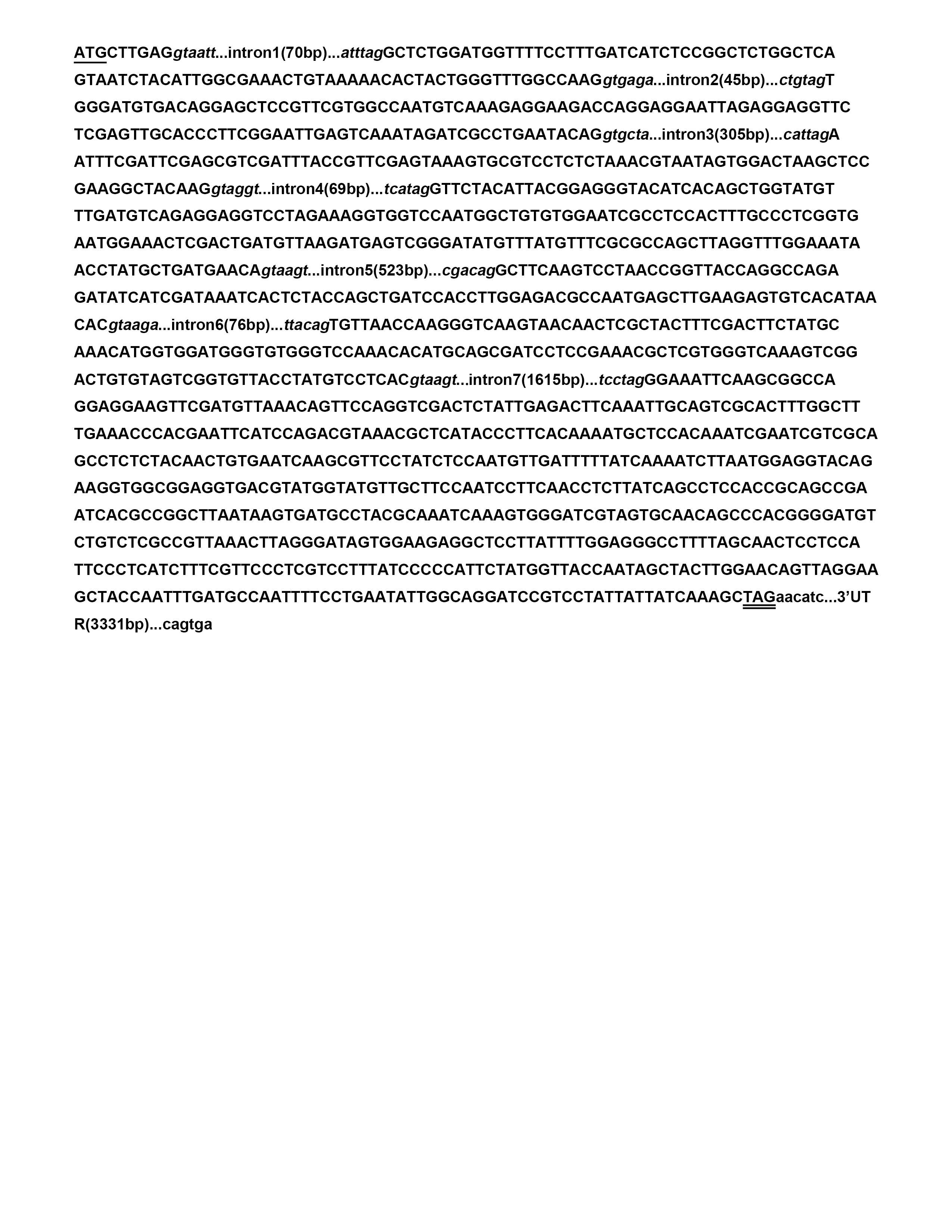

Supplement: SUPPLEMENTARY FIGURE S3 — Gene sequence of emhif1α. Introns were identified by comparing the genomic DNA sequence with cDNA. The length of each intron and its partial sequence including the canonical GT-AG-dinucleotides at the 5’ splice donor and the 3’ splice acceptor sites (italic lowercase) are given. The translational start codon is indicated by a single line “—”. The stop codon is indicated by a double line “”. Sequences of 3’ untranslated regions are shown in lowercase. Coding sequence is shown in uppercase. Note that the actual size (1,347 bp) of the emhif1α smaller than predicted (1,839 bp). The emhif1α gene spans a genomic region of 7,381 kb and comprised 8 exons, separated by 7 introns. [file Image_3.TIF]

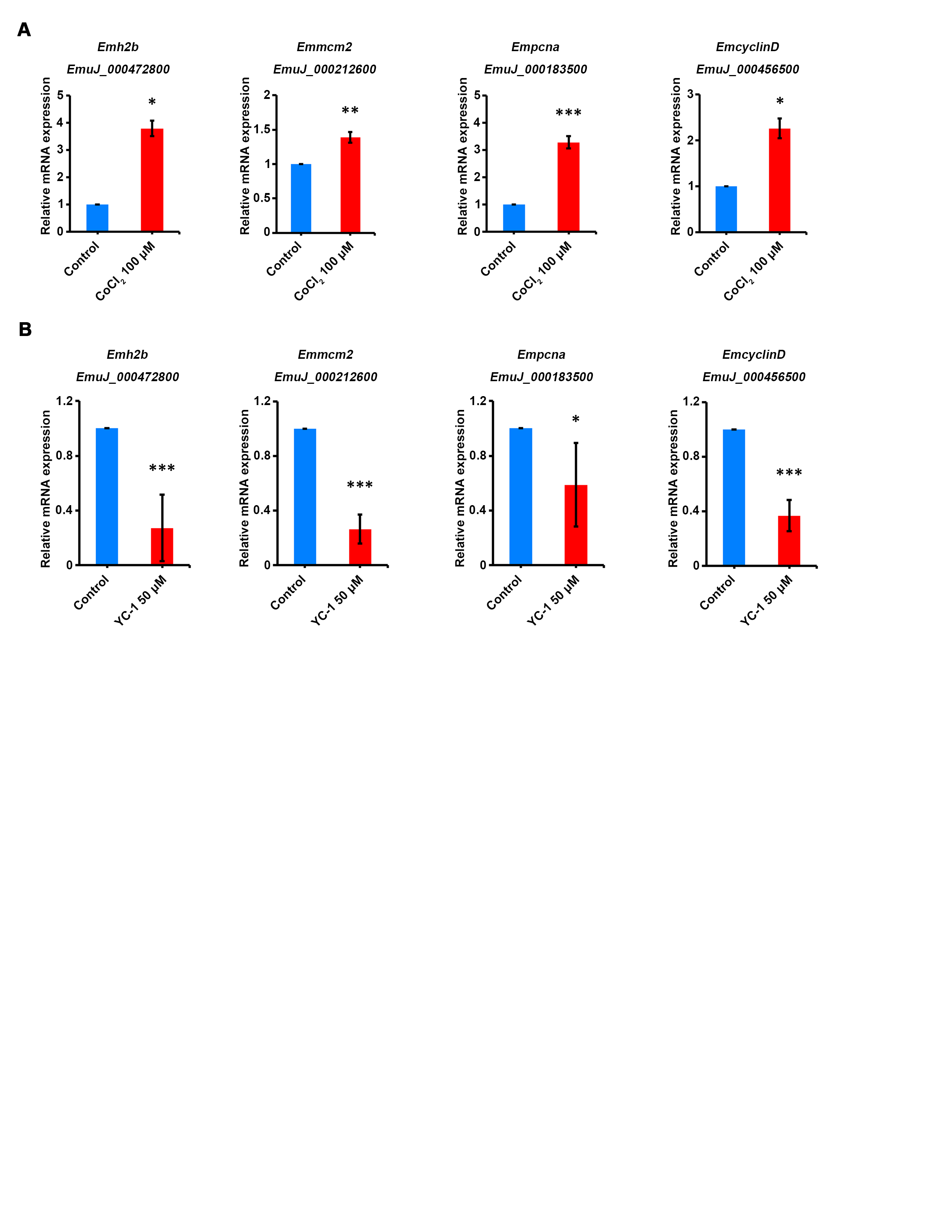

Supplement: SUPPLEMENTARY FIGURE S4 — EmHIF1α agonist and inhibitor regulate the expression of cell cycle-related genes in metacestode vesicles. Real-time PCR analysis of the mRNA expressions of cell cycle-related factors in the metacestode vesicles treated with 100 μM CoCl2 (A) or 50 μM YC-1 for 4 hours (B). Data were shown as mean ± SD. The significance was determined by student’ s t-test. *p < 0.05, **p < 0.01, ***p < 0.001. [file Image_4.TIF]

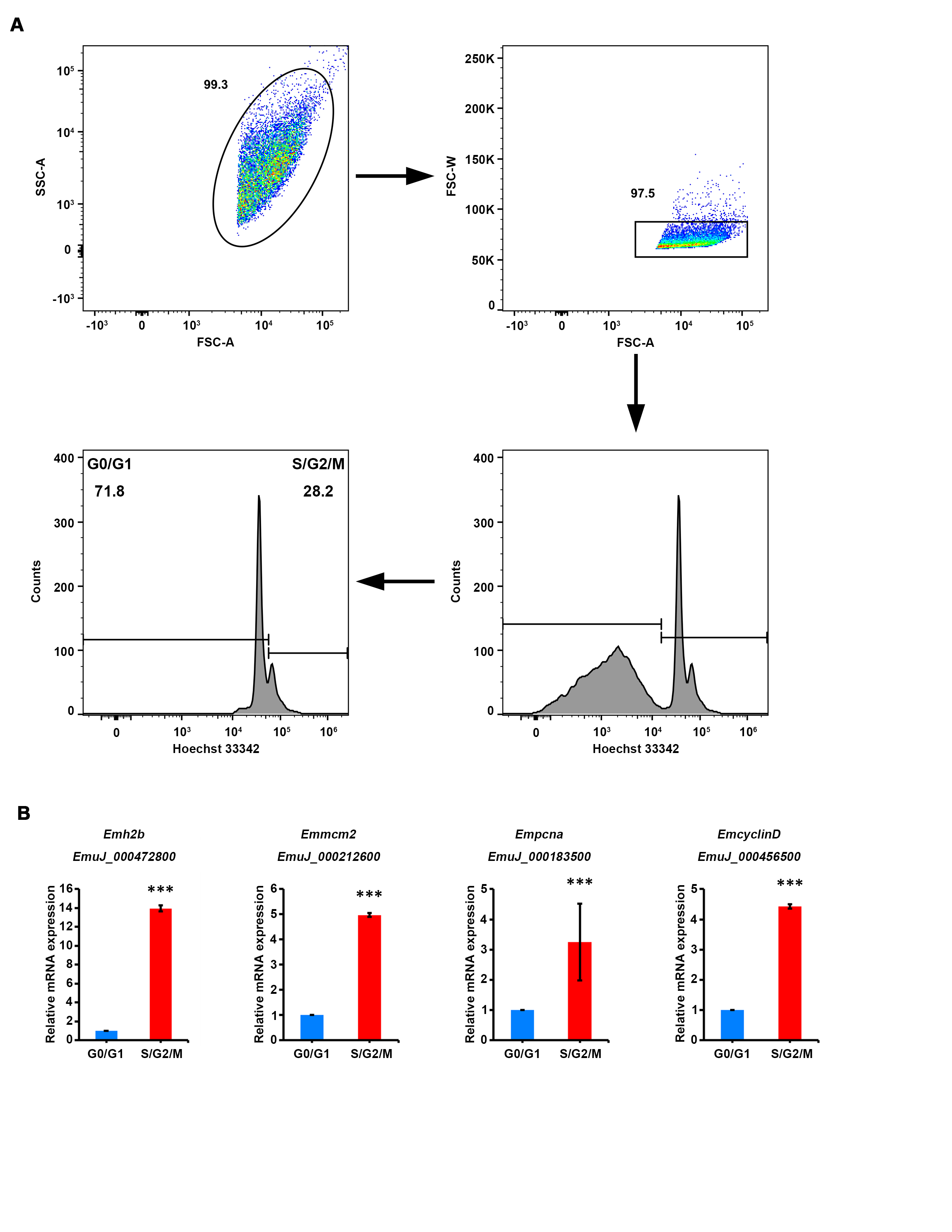

Supplement: SUPPLEMENTARY FIGURE S5 — Flow cytometry sorting strategy. (A) Gating strategy for the primary cells freshly isolated from metacestode vesicles. Upper left: chief cell population (black oval), upper right: chief cell population after removing adhesions (black rectangle), lower right: chief cell population after removing fragmented fluorescence (right line segment), lower left: DNA content of the primary cells. (B) Freshly isolated primary cells were stained with Hoechst 33342 for sorting based on DNA content. mRNA expressions of a set of cell cycle-related factors were analyzed after cell sorting. Data in (B) were shown as mean ± SD. The significance was determined by student’ s t-test. ***p < 0.001. [file Image_5.TIF]
