# Supplementary material for: ROS are required for the germinative cell proliferation and metacestode larval growth of Echinococcus multilocularis
Source: Front Microbiol. 2024 Jun 7;15:1410504. doi: 10.3389/fmicb.2024.1410504 (PMC11190091; doi:10.3389/fmicb.2024.1410504)
Supplement: Supplementary file 6 [file Table_1.DOCX]

**Table S1. The accession numbers of HIF1α and HIF1β for alignment and phylogenetic tree construction.**

| **Species** | **Protein Name** | **Accession Number (UniProt)** |
| --- | --- | --- |
| ***Echinococcus multilocularis*** | Em-HIF1α | A0A068Y8C6 |
|  | Em-HIF1β | A0A068YE51 |
| ***Caenorhabditis elegans*** | Ce-HIF1α | G5EGD2 |
|  | Ce-HIF1β | O02219 |
| ***Homo sapiens*** | Hs-HIF1α | Q16665 |
|  | Hs-HIF1β | P27540 |
| ***Mus musculus*** | Mm-HIF1α | Q61221 |
|  | Mm-HIF1β | P53762 |
| ***Drosophila melanogaster*** | Dm-HIF1α | Q24167 |
|  | Dm-HIF1β | O15945 |
| ***Danio rerio*** | Dr-HIF1α | Q6EHI4 |
|  | Dr-HIF1β | Q9DG12 |
| ***Xenopus laevis*** | Xl-HIF1α | Q9I8A9 |
|  | Xl-HIF1β | Q90X20 |

The login URL for obtaining protein sequences is https://www.uniprot.org/.
