# Supplementary material for: ROS are required for the germinative cell proliferation and metacestode larval growth of Echinococcus multilocularis
Source: Front Microbiol. 2024 Jun 7;15:1410504. doi: 10.3389/fmicb.2024.1410504 (PMC11190091; doi:10.3389/fmicb.2024.1410504)
Supplement: Supplementary file 7 [file Table_2.DOCX]

**Table S2. Primers used in this study.**

| **Purposes** | **Primer Name** | **Sequence (5’ > 3’)** | **Reference** |
| --- | --- | --- | --- |
| **Identification of** ***EmHIF1α*** | *EmHif1α*-F | ATGCTTGAGGCTCTGGATGGTTTTC | This study |
|  | *EmHif1α*-R | TGCAACCTTGTGTGCTTCATTGACA | This study |
| **3’ RACE** | GSP | GGAATCGCCTCCACTTTGCCCTCGGT | This study |
|  | NUP | AAGCAGTGGTATCAACGCAGAGT | This study |
| **mRNA expression analysis** | *Emh2b*-qF | TAAGAAGAAGAGGAGGAAG | This study |
|  | *Emh2b*-qR | TAGACTCAGCGGCAAT | This study |
|  | *Emmcm2*-qF | ACCGTAAATGAGTGGG | This study |
|  | *Emmcm2*-qR | TCGGGAAGGAAGTAAG | This study |
|  | *Empcna*-qF | AAGCCGATGTATGGAAGA | This study |
|  | *Empcna*-qR | AAGAGGGAAACAAGACTGA | This study |
|  | *EmcyclinD*-qF | GGAATTGTTTGCAAAAGAGC | [S(Feng et al., 2022)] |
|  | *EmcyclinD*-qR | GATCCTCGAATAAGCTGCTG | [S(Feng et al., 2022)] |
|  | *elp*-qF | CAGGATCTCTTCGATCAAGTG | [S(Brehm et al., 2003)] |
|  | *elp*-qR | GACCATACTTGGCAACACAGG | [S(Brehm et al., 2003)] |
| **si*EmHIF1α*** | 7013 | CGGAGATACTCACCACTAT | This study |
|  | 8053 | TCACTTGCACTCCTCTTCA | This study |
|  | 9063 | GGACATCACCAACCAATTC | This study |

**References:**

Brehm, K., Wolf, M., Beland, H., Kroner, A., and Frosch, M. (2003). Analysis of differential gene expression in Echinococcus multilocularis larval stages by means of spliced leader differential display. Int J Parasitol 33(11). doi: 10.1016/s0020-7519(03)00169-3.

Feng, C., Cheng, Z., Xu, Z., Tian, Y., Tian, H., Liu, F., et al. (2022). EmCyclinD-EmCDK4/6 complex is involved in the host EGF-mediated proliferation of Echinococcus multilocularis germinative cells via the EGFR-ERK pathway. Frontiers in Microbiology 13. doi: 10.3389/fmicb.2022.968872.
